# Physical activity and kidney diseases

**DOI:** 10.1186/1824-7288-40-S1-A21

**Published:** 2014-08-11

**Authors:** Silvio Maringhini

**Affiliations:** 1 Pediatric Nephrology Unit. “G. Di Cristina” Children’s Hospital. A.R.N.A.S. Palermo. Via Benedettine 2, 90134 Palermo, Italy

## 

Regular physical activity (PA) in the early school years is recommended by several scientific associations for primary prevention of cardiovascular disease. Long-term observational studies have shown that subjects who exercise regularly have significantly less coronary heart disease (CHD) and a reduced risk of cardiovascular disease (CVD). Exercise reduces serum triglycerides, increases serum high density lipoprotein-cholesterol, lowers the blood pressure in patients with primary hypertension [1]. Regular exercise reduces the production of atherogenic cytokines and increases production of atheroprotective cytokines. It should be mentioned, however, that other factors may be associated with physical activity (e.g. a healthy diet, avoiding cigarette smoking, regular medical care) and may contribute to the improved health. No long term studies have been produced on the effects of PA during childhood on CHD and CVD in adult life but indirect evidence suggests that it may produce benefit.

Regular exercise, on the other end, is associated with potential adverse effects (eg, musculoskeletal injuries, arrhythmias, myocardial infarction, and rhabdomyolysis); however, the absolute risk of kidney disease during exercise is low [2]. Consensus guidelines for the pre-participation physical evaluation (PPE) suggest a PPE for all children, even those who do not participate in organized sports, as an opportunity to promote health and fitness. PPE includes a medical and family history and a physical examination, with particular emphasis on the musculoskeletal and cardiovascular systems [3].

Exercise induces profound changes in the renal haemodynamics and in electrolyte and protein excretion. Proteinuria, hematuria and changes in serum electrolyte balance have been reported during intense PA; the increase in glomerular filtration may explain these transient alterations but the “nutcracker” compression on the renal vein may have a role. Haemoglobinuria and myoglobinuria may be observed under special exercise conditions [4].

PA does not worse nor reverse kidney disease but may reduce cardiovascular risk in chronic renal disease [5]. Children on dialysis and after a renal transplantation performing aerobic physical exercise show improvements in exercise tolerance, in quality of health and uraemic symptom scores; they gain weight loss, cardiovascular reactivity, avoiding an increase in blood pressure medication [6].
